# A Review on Umbilical Cord Milking and Its Implications in Neonatal Health

**DOI:** 10.7759/cureus.30610

**Published:** 2022-10-23

**Authors:** Shubhi N Jain, Ashok M Mehendale

**Affiliations:** 1 College of Medicine, Jawaharlal Nehru Medical College, Datta Meghe Institute of Medical Sciences, Wardha, IND; 2 Community Medicine, Jawaharlal Nehru Medical College, Datta Meghe Institute of Medical Sciences, Wardha, IND

**Keywords:** infant, late cord clamping, early cord clamping, infant mortality rate, umbilical cord milking

## Abstract

In India, there is an extreme lack of advancement in techniques concerning the care of infants during labor and post-pregnancy, i.e., the postnatal period. India projected an Infant Mortality Rate of 28.771 deaths per 1000 live births for the year 2021. Such a high death rate in infants arises a dire need to discover and launch new techniques and increase the application of the existing lesser-known techniques. One less well-known technique is the milking of the infant's umbilical cord. Transfusion of placental blood has recently been considered definitive in the care of newborns with the boon to the infant of declined death rate in preterm neonates and the enhanced consequences of growth in term infants. The chief goal of this descriptive review article is to examine all the studies relating to umbilical cord milking (UCM) in late-preterm and term infants and to evaluate every achievable outcome and restriction of a given process in clinical application, mainly when compared to rapid and late umbilical cord clamping. Application of milking of the umbilical cord can be seen to improve the health of hypoxic neonates, weight parameters, blood volume, hematocrit, hemoglobin, iron levels in the blood, red blood cell count, blood pressure, right ventricular output, left ventricular functions, cerebral oxygenation, urine output regulation, cognitive abilities, antioxidant levels, better outcomes in the resuscitation of infant and above all helps in lowering Infant Mortality Rates.

## Introduction and background

Following the birth of an infant, the volume of blood flowing in the umbilical veins and arteries typically carries on for some minutes. The supplemental volume of blood transferred to the infant throughout this time is called “Placental transfusion” [1]. At the moment of delivery of the baby, there is a transformation in the infant, from depending on the blood through the umbilical cord for oxygen to beginning to respire through their lungs. While the lungs ask for the blood, this transformation activates changes in the blood flow of the infant.

Previous to the mid of 1950s, the name early clamping referred to the clamping of the umbilical cord in less than one minute of birth, and late clamping referred to the clamping of the umbilical cord taking time beyond five minutes post birth of the baby. In a string of various microscale surveys of change in blood volume post-birth, it was noticed that in healthy babies, 90% of the volume of blood transfusion takes place in the initial 2-3 breaths [2], and in the initial three minutes of the birth, transfer of around 80 to 100 milliliters of the volume of blood through the placenta into the newly born baby occurs [2-4].

In the process of milking from the umbilical cord, the umbilical cord, either unclamped or clamped, is clasped; and the blood in it is propelled 3 to 4 times to the side of the newborn. It is usually a speedy process expected to be done in less than 20 secs. The main objective of milking the umbilical cord is to supply neonates with the maximum possible volume of blood- the volume which is generally lacking as in the case of early cord clamping (which is the completion of transfusion of placental blood in a time not more than taken for late clamping of cord).

The chief goal of this descriptive review article is to examine all the studies relating to UCM in late-preterm and term infants and to evaluate every achievable outcome and restriction of a given process in clinical application, mainly when compared to rapid and late umbilical cord clamping [5]. Due to these before-time observations and the deficiency of particular suggestions with reference to optimal timing, the mean time between the delivery of the baby and the clamping of the umbilical cord; began to be reduced, and it now has become an ordinary exercise to clamp the umbilical cord rapidly post birth, generally within the time of 15 to 20 seconds. Nevertheless, more up-to-date random controlled trials relating to preterm and term neonates, along with the study of the physiology of blood volume, arterial pressure, and oxygenation, have estimated the outcomes of early vs. delayed umbilical cord clamping (which is generally defined as clamping of the umbilical cord in less than 30 to 60 seconds post-birth) [6,7].

The worldwide application of late umbilical cord clamping has elevated apprehension. In case of resuscitation required, mainly in preterm babies, delayed clamping of the umbilical cord can cause hindrance in timely attempts for resuscitation. But, as the placenta keeps carrying out the exchange of gas post-delivery, ill and premature neonates are probably to advantage in the majority from the supplementary volume of blood obtained from the continuous exchange from the placenta.

UCM is becoming more popular as a secure, efficient, and advantageous substitute for late cord clamping, particularly for premature newborns, as well as those born through cesarean section. Contrary to delayed cord clamping (DCC), UCM imparts a transfusion of the placental blood without putting off resuscitation and can be finished as soon as early cord clamping [8].

UCM allows transfusion of placental blood by pushing blood towards the newly born before the clamping of the umbilical cord with a time duration equivalent to that of immediate umbilical cord clamping letting the pediatric team begin with the resuscitation immediately [9,10].

## Review

Types of milking umbilical cord

UCM can be performed in two ways: Intact umbilical cord milking (I-UCM) and cut-umbilical cord milking (C-UCM). I-UCM (intact umbilical cord milking) is termed when milking is done in the cord, which is attached to the placenta even after delivery of the baby. Furthermore, C-UCM (cut-umbilical cord milking) is termed when milking is done in the cord, which is cut and removed from the placenta [5].

Intact UCM stands as a substitute for delayed cord clamping once the unclamped umbilical cord is held and blood is propelled or stripped around 2 to 4 times before being clamped. Vigilant observation must be made on how the process of cord milking is carried out, that is, if the cord is held intact, along with how many times it should be done. Various studies reveal that sudden improvement in the blood flow of the lung (pulmonary blood flow) and support in the expansion of the lungs during the initiation of breathing can be observed with an intact cord [2].

In contrast to this, C-UCM, which is primarily practiced in the continent of Asia, includes clamping and then cutting cord segment at a distance of 25 cm from the umbilical stump instantly after delivery of the baby. The long cord is then taken by a pediatric practitioner; he uncoils it and milks the whole contents into the infant [11].

Steps of umbilical cord milking

In infants who underwent unmutilated UCM, with the use of one hand, the cord is squeezed and held nearest to the end of the placental side. The other hand is utilized for milking blood in the direction of the neonate, where the cord is held at the umbilical endpoint. The method mentioned is named one-sided milking motion.

This cord is afterward freed at the end with the placenta and permitted to be refilled over 1 to 2 seconds in between every motion of milking. The mentioned process is to be repeated four times in total. Following the milking, the cord is supposed to be clamped and then cut. Further, the infant is given to the pediatric team to take further care of the baby [12].

The term infant received anywhere between 50 and 100 milliliters of blood when the milking procedure was utilized, albeit the volume differed [13].

Advantages of umbilical cord milking

In Hypoxic Neonates

The marked benefit is provided by UCM over delayed cord clamping (DCC) in newly born babies, which are hypoxic. These oxygen-deficient infants cannot be expected to wait for delayed cord clamping because they are at a greater risk of developing life-threatening intraventricular bleeding or even death [1,13].

Cognitive Domain

Neonates surveyed for UCM have observed better linguistic skills and logical reasoning scores compared with those surveyed for DCC. There were no variations in the grade of mild to moderate or severe neurodevelopmental disablement [14].

Blood Volume

UCM is considered to be an extra skillful method to raise the volume of blood in premature neonates who are birthed by caesarean delivery [15,16].

Weight Parameters

The neonates born using UCM had higher birth weights, decreased weight loss, and a slower rate of weight reduction [13,17].

Blood Pressure and Urine Output

The blood pressure of the UCM group was substantially more significant for the initial 12 hours. Throughout the initial 72 hours, the UCM group's urine output was more compared to that of the control group. In newborns with extremely low birth weight, UCM can help with the timely regulation of both urine output and blood pressure. No major variation in levels of the ventilatory index, water consumption and pulse rate was noted among the UCM and control groups [18].

Hematologic Parameters

A systemic review exhibited a comparison of milking of the umbilical cord (MUC) with rapid clamping of the umbilical cord (RCUC). The study revealed that MUC was linked with increased values of hematocrit and hemoglobin, reduced risk of the requirement of oxygen, and a reduced risk of all levels of intraventricular hemorrhage at a gestational age of 36 weeks in premature neonates [9,19]. The method of umbilical cord clamping is generally known to give around 30% of the extra volume of blood and 60% of extra red blood cells (RBC) to the newborn as compared to immediate clamping of the cord [20,21].

Amongst the newborns with a minimal gestational age of 33 weeks, UCM was linked with increased hemoglobin values in 224 infants in the initial 48 hours of their birth and in 170 infants at six weeks of life [19,22]. No trial to date has exhibited any impairment associated with UCM.

Right Ventricular Output

In a study carried out in the year 2014, the enrolled number of neonates was 60 who were randomized, out of which 30 neonates were allocated to immediate cord clamping and the rest 30 neonates to cord milking. Neonates surveyed on cord milking had more special estimates of correct ventricular output and superior vena cava flow in the initial six hours and 30 hours of life.

Newborns getting UCM also were seen to receive lesser blood transfusions, higher serum hemoglobin, a less frequent need for oxygen, and a shorter duration of oxygen supplementation at the corrected postmenstrual age of 36 weeks [23].

Left Ventricle Function

The flow of the superior vena cava, cardiac output of the left ventricle, end-diastolic dimension of the left ventricle and hematocrit were observed to be greater in value in the group of cord milking in comparison to the group control. Also, LV Tei Index advancement is marked. LV Tei Index is used worldwide for measurement of the function of the left ventricle, which is estimated using the transoesophageal echocardiogram (TEE) [24]. Even after the deprivation of changes in the ejection fraction of the left ventricle, in less than 24 hours post-birth, LV Tei Index advancement was observed [25].

**Figure 1:**
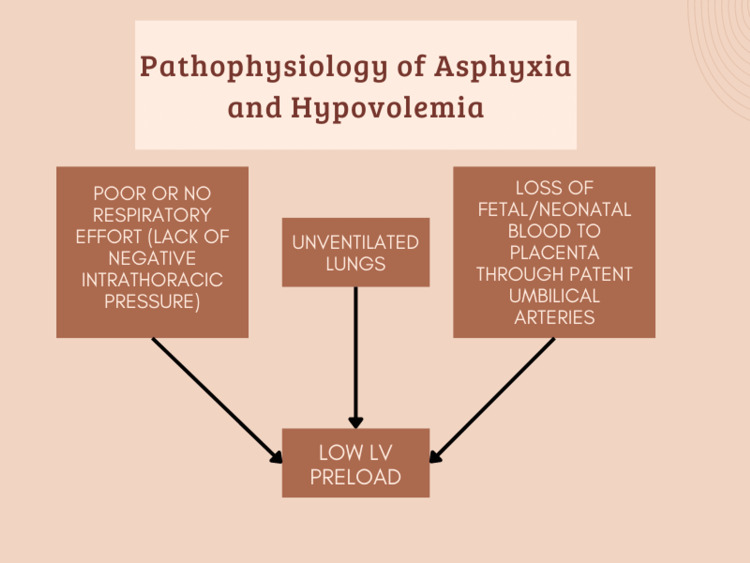
Pathophysiology of Asphyxia and Hypovolemia [15]

Figure 1 shows pathophysiology of asphyxia and hypovolemia.

Cerebral Oxygenation

The tissue oxygenation index increased, and cerebral fractional tissue oxygen extraction decreased in the milked group within 24 hours after birth [25].

In Depressed Neonates

Milking of the umbilical cord is practicable for neonates who are depressed at the moment of birth, be it late preterm neonates or term neonates. Among the two groups of UCM and the control group, there were no discernible changes in resuscitation efforts, short-term results and resuscitation delay. For infants with HIE (Hypoxic Ischemic Encephalopathy), a more significant clinical study is required for the assessment of the long-term advantages of UCM [26].

Antioxidant Levels

A randomized study carried out in the year 2016 aimed to examine the balance of thiol-disulphide in three different methods of clamping the cord, i.e., cord milking, delayed clamping and early clamping, to comment on their antioxidant levels. The sample size of this survey was 189 full-term neonates who were fractioned into three sections based on the method of clamping their cord: cord milking delayed clamping and early clamping. The homeostasis of thiol/disulphide was examined using the sample of blood which was taken through the umbilical arteries shortly after clamping. Blood samples were collected from the umbilical arteries immediately after clamping, and the thiol/disulphide homeostasis was analyzed.

Cord milking and delayed cord clamping shows a markedly reduced ratio of disulphide to total thiol in contrast to the group of immediate cord clamping. In the group of immediate cord clamping, considerably decreased total along with aboriginal amounts of thiol were recorded when set side by side with cord milking and delayed clamping of the cord. When contrasted to the process of cord milking and late cord clamping, early cord clamping results in the creation of decreased levels of thiol and increased number of disulphide bonds, suggesting that oxidation reactions are intensified. When the cord is clamped early as opposed to when it is clamped later or when the cord is milked, a marked rise in the oxidant capacity is observed. We advise that cord milking or late cord clamping be done frequently during every childbirth because they are proven to be advantageous for neonatal health [27].

Iron Levels

Maintaining the umbilical cord long as well as milking it could be a successful strategy for increasing hemoglobin and iron reserves in term newborns at the age of six months [28,29].

Reduced Infant Mortality

Transfusion of placental blood has recently been considered definitive in the care of the newborn with the benefit to the infant of the declined death rate in preterm neonates in addition to the enhanced outcomes of growth in term infants [9,30,31]. Delayed umbilical cord clamping has been replaced by UCM, which is considered a recent definitive of neonatal care.

Others

In a study carried out, 318 ELGANs (Extremely Low Gestational Age Newborns) were identified, out of which 158 neonates were competent for UCM (Umbilical Cord Milking) along with the rest 160 neonates were competent for retrospective control. No harmful happenings were noted during the process of milking the cord.

In spite of less vasopressor used, improved hemodynamic stability in the initial one day of neonatal age was observed to be the increased value of Mean Blood Pressure in the UCM group. Compared to the control group, decreased red cell transfusions and increased value of initial hematocrit were observed in UCM.

Decreased cases of necrotizing enterocolitis and intraventricular hemorrhage were reported. Even reduction in mortality prior to discharge from hospital [32].

UCM decreases the demand for transfusion of RBCs and for pulmonary and circulatory assistance in preterm neonates and is thus considered a safe technique [33,34]. 

**Figure 2:**
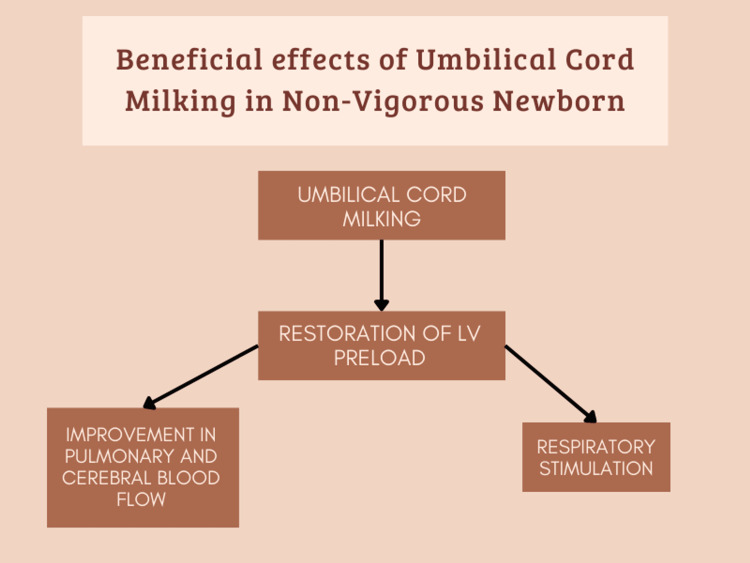
Beneficial Effects of Umbilical Cord Milking in Non-Vigorous Newborn [15]

Figure 2 shows the beneficial effects of umbilical cord milking in non-vigorous newborn. 

Disadvantages of umbilical cord milking

Neutrophil Count

A clinical trial was carried out in 2014 with a sample size of 58 pregnant women. This clinical trial aimed to estimate the effect of UCM on the neutropenia frequency and the (ANCs) absolute neutrophil counts of preterm neonates. Fifty-eight pregnant women were allocated randomly to either the UCM group or the control group. In total, 54 infants of preterm age, i.e., gestational age ≤32 weeks, were enrolled on the research. Twenty-five neonates had their umbilical cords clamped as soon as they were born, while 29 neonates had their umbilical cords initially milked. Experimentally, on days one, three and seven, the levels of ANCs were remarkably reduced in the group of cord milking in contrast to the control group.

The control group showed a decreased prevalence of neutropenia in contrast to the UCM group [35].

Multiple-Time Milking

In extremely premature neonates, it is observed that mostly cutting the umbilical cord and milking it just one time show the same result as milking the intact umbilical cord multiple times [36].

## Conclusions

This review article focuses on a simple approach that has the potential to enhance perinatal outcomes by milking the umbilical cord of premature newborns. The main aim of the article was to study the benefits of UCM over immediate cord clamping in infants to reduce the infant mortality rate, which is a major health drawback in the country. Application of milking of the umbilical cord can be seen to improve the health of hypoxic neonates, weight parameters, blood volume, hematocrit, hemoglobin, iron levels in the blood, red blood cell count, blood pressure, right ventricular output, left ventricular functions, cerebral oxygenation, urine output regulation, cognitive abilities, antioxidant levels, better outcomes in the resuscitation of infant and above all helps in lowering Infant Mortality Rates. The article tried to brief why the process of UCM should be more common in India. We tried to study numerous articles and gather relevant information and compile it in the best possible way. 

## References

[1] (2022). Royal College of Obstetricians and Gynaecologists: Clamping of the umbilical cord and placental transfusion (scientific impact paper no 14). https://www.rcog.org.uk/guidance/browse-all-guidance/scientific-impact-papers/clamping-of-the-umbilical-cord-and-placental-transfusion-scientific-impact-paper-no-14/..

[2] (2017). Committee opinion no. 684: Delayed umbilical cord clamping after birth. Obstet Gynecol.

[3] Yao AC, Moinian M, Lind J (1969). Distribution of blood between infant and placenta after birth. Lancet.

[4] Jeevan A, Ananthan A, Bhuwan M, Balasubramanian H, Rao S, Kabra NS (2021). Umbilical cord milking versus delayed cord clamping in term and late-preterm infants: a systematic review and meta-analysis. J Matern Fetal Neonatal Med.

[5] Basile S, Pinelli S, Micelli E, Caretto M, Benedetti Panici P (2019). Milking of the umbilical cord in term and late preterm infants. Biomed Res Int.

[6] Rabe H, Diaz-Rossello JL, Duley L, Dowswell T (2012). Effect of timing of umbilical cord clamping and other strategies to influence placental transfusion at preterm birth on maternal and infant outcomes. Cochrane Database Syst Rev.

[7] McDonald SJ, Middleton P, Dowswell T, Morris PS (2013). Effect of timing of umbilical cord clamping of term infants on maternal and neonatal outcomes. Cochrane Database Syst Rev.

[8] Katheria AC (2018). Umbilical cord milking: A review. Front Pediatr.

[9] Katheria A, Reister F, Essers J (2019). Association of umbilical cord milking vs delayed umbilical cord clamping with death or severe intraventricular hemorrhage among preterm infants. JAMA.

[10] Mercer JS (2001). Current best evidence: a review of the literature on umbilical cord clamping. J Midwifery Women's Health.

[11] Upadhyay A, Gothwal S, Parihar R, Garg A, Gupta A, Chawla D, Gulati IK (2013). Effect of umbilical cord milking in term and near term infants: randomized control trial. Am J Obstet Gynecol.

[12] Sura M, Osoti A, Gachuno O (2021). Effect of umbilical cord milking versus delayed cord clamping on preterm neonates in Kenya: A randomized controlled trial. PLoS One.

[13] McCausland AM, Holmes F, Schumann WR (1949). Management of cord and placental blood and its effect upon the newborn. Calif Med.

[14] Katheria A, Garey D, Truong G (2018). A randomized clinical trial of umbilical cord milking vs delayed cord clamping in preterm infants: Neurodevelopmental outcomes at 22-26 months of corrected age. J Pediatr.

[15] Katheria AC, Truong G, Cousins L, Oshiro B, Finer NN (2015). Umbilical cord milking versus delayed cord clamping in preterm infants. Pediatrics.

[16] McAdams RM, Fay E, Delaney S (2018). Whole blood volumes associated with milking intact and cut umbilical cords in term newborns. J Perinatol.

[17] Siddall RS, Crissey RR, Knapp WL (1952). Effect on cesarean section babies of stripping or milking of the umbilical cords. Am J Obstet Gynecol.

[18] Hosono S, Mugishima H, Fujita H (2009). Blood pressure and urine output during the first 120 h of life in infants born at less than 29 weeks' gestation related to umbilical cord milking. Arch Dis Child Fetal Neonatal Ed.

[19] Al-Wassia H, Shah PS (2015). Efficacy and safety of umbilical cord milking at birth: a systematic review and meta-analysis. JAMA Pediatr.

[20] Song SY, Kim Y, Kang BH, Yoo HJ, Lee M (2017). Safety of umbilical cord milking in very preterm neonates: a randomized controlled study. Obstet Gynecol Sci.

[21] Fogarty M, Osborn DA, Askie L (2018). Delayed vs early umbilical cord clamping for preterm infants: a systematic review and meta-analysis. Am J Obstet Gynecol.

[22] Erickson-Owens DA, Mercer JS, Oh W (2012). Umbilical cord milking in term infants delivered by cesarean section: a randomized controlled trial. J Perinatol.

[23] Katheria AC, Leone TA, Woelkers D, Garey DM, Rich W, Finer NN (2014). The effects of umbilical cord milking on hemodynamics and neonatal outcomes in premature neonates. J Pediatr.

[24] Sivanandam S, Wey A, St Louis J (2015). Intraoperative transesophageal echocardiographic assessment of left ventricular Tei index in congenital heart disease. Ann Card Anaesth.

[25] Takami T, Suganami Y, Sunohara D (2012). Umbilical cord milking stabilizes cerebral oxygenation and perfusion in infants born before 29 weeks of gestation. J Pediatr.

[26] Girish M, Jain V, Dhokane R, Gondhali SB, Vaidya A, Aghai ZH (2018). Umbilical cord milking for neonates who are depressed at birth: a randomized trial of feasibility. J Perinatol.

[27] Vatansever B, Demirel G, Ciler Eren E (2018). Is early cord clamping, delayed cord clamping or cord milking best?. J Matern Fetal Neonatal Med.

[28] Bora R, Akhtar SS, Venkatasubramaniam A, Wolfson J, Rao R (2015). Effect of 40-cm segment umbilical cord milking on hemoglobin and serum ferritin at 6 months of age in full-term infants of anemic and non-anemic mothers. J Perinatol.

[29] Agarwal S, Jaiswal V, Singh D, Jaiswal P, Garg A, Upadhyay A (2016). Randomised control trial showed that delayed cord clamping and milking resulted in no significant differences in iron stores and physical growth parameters at one year of age. Acta Paediatr.

[30] Tarnow-Mordi W, Morris J, Kirby A (2017). Delayed versus immediate cord clamping in preterm infants. N Engl J Med.

[31] Andersson O, Lindquist B, Lindgren M, Stjernqvist K, Domellöf M, Hellström-Westas L (2015). Effect of delayed cord clamping on neurodevelopment at 4 years of age: A randomized clinical trial. JAMA Pediatr.

[32] Patel S, Clark EA, Rodriguez CE, Metz TD, Abbaszadeh M, Yoder BA (2014). Effect of umbilical cord milking on morbidity and survival in extremely low gestational age neonates. Am J Obstet Gynecol.

[33] Hosono S, Mugishima H, Fujita H (2008). Umbilical cord milking reduces the need for red cell transfusions and improves neonatal adaptation in infants born at less than 29 weeks' gestation: a randomised controlled trial. Arch Dis Child Fetal Neonatal Ed.

[34] Katheria A, Mercer J, Brown M (2018). Umbilical cord milking at birth for term newborns with acidosis: neonatal outcomes. J Perinatol.

[35] Kilicdag H, Gulcan H, Hanta D, Torer B, Gokmen Z, Ozdemir SI, Antmen BA (2016). Is umbilical cord milking always an advantage?. J Matern Fetal Neonatal Med.

[36] Hosono S, Mugishima H, Takahashi S, Takahashi S, Masaoka N, Yamamoto T, Tamura M (2015). One-time umbilical cord milking after cord cutting has same effectiveness as multiple-time umbilical cord milking in infants born at &lt;29 weeks of gestation: a retrospective study. J Perinatol.

